# Supplementary material for: The role of OLE2 and POX1-3 in prostaglandin E2 production and virulence is conserved in Candidozyma (Candida) auris
Source: Microbiol Spectr. 2026 Jan 28;14(3):e02323-25. doi: 10.1128/spectrum.02323-25 (PMC12955410; doi:10.1128/spectrum.02323-25)
Supplement: Supplemental material — Tables S1 to S4, Figures S1 to S12, and supplemental Materials and Methods. [file spectrum.02323-25-s0001.docx]

**Materials and Methods**

**Primer Design for the CRISPR cas9 gene editing system**

Primer design for the CRISPR-Cas9 system was performed using Geneious Prime® 2023.0.1 ([www.geneious.com](http://www.geneious.com/)) and purchased from Integrated DNA Technologies (Table S1)

**Table S1.** Primers used to develop the CRISPR-Cas9 system

| Primer name | Primer sequence (5' – 3') | Tm (˚C) | GC% | Length (bp) |
| --- | --- | --- | --- | --- |
| AHO1237 | AGGTGATGCTGAAGCTATTGAAG | 58.8 | 43.5 | 23 |
| Cau-snR52-1R | GTTTTCTGCTGAGGGAGTC | 55.5 | 52.6 | 19 |
| Generic-addback-gRNA | CGTAAACTATTTTTAATTTGGTAGCAACTGGAATTCCTCGGTTTTAGAGCTAGAAATAGC | 72.8 | 38.3 | 60 |
| Cau-HIS1-2R | TAACACTCTATTTCAGAAATGTCG | 55.0 | 33.3 | 24 |
| *OLE2* -1F (AUX) | AGATGATGCTTGCACGGACT | 59.7 | 50.0 | 20 |
| *OLE2*-1F | GGCAGACTGGTGATCTCGAAA | 60.1 | 52.4 | 21 |
| *OLE2*-3F | TGCTCGCATCAATGATATGAGGA | 60.0 | 43.5 | 23 |
| *OLE2*-4R CRISPR Site | CCTCGAGGAATTCCAGTTGCTACTGATACCCAGCAGTGAAGGC | 68.8 | 53.5 | 43 |
| *OLE2*-4F CRISPR site | GTAGCAACTGGAATTCCTCGAGGAGGCGCAGAAGTCAACTCAA | 68.8 | 51.2 | 43 |
| *OLE2*-3R | ACAGCAGCAGTCAGATGTCC | 60.0 | 55 | 20 |
| *OLE2*-1R | TAGTGCTTACCCTTGCCTGC | 60.0 | 55.0 | 20 |
| *OLE2*-1R (AUX) | GGTCGCGGTCATCATTGAAC | 59.6 | 55.0 | 20 |
| *OLE2* screen (AUX) | TTGTCAGAGATGAAGAAGGC | 55.1 | 45.0 | 20 |
| *POX1-3* 1F (AUX) | TCAGTCGACTTACCAGCAGC | 59.8 | 55.0 | 20 |
| *POX1-3*-1F | GCCGACAAGTGAGCAAGAAC | 59.8 | 55.0 | 20 |
| *POX1-3* -3F | GGAGACCGCTGAAAAGTCGA | 60.0 | 55.0 | 20 |
| *POX1-3* -4R CRISPR Site | CCTCGAGGAATTCCAGTTGCTACCAAGCACACTCAAGAGCGAC | 69.2 | 53.5 | 43 |
| *POX1-3* -4F CRISPR Site | GTAGCAACTGGAATTCCTCGAGGCGAAACCAACGAGCACAAGG | 69.7 | 53.5 | 43 |
| *POX1-3* -3R | GAACGAGTCGGTGTAGGCAA | 60.0 | 55.0 | 20 |
| *POX1-3*-1R | ATGGTCTTGCATACCCCACG | 60.1 | 55.0 | 20 |
| *POX1-3* -1R (AUX) | GAGCTCGTAGTCCCGGAAAG | 59.9 | 60.0 | 20 |
| *POX1-3* screen (AUX) | ATCAACTGGTGTCTTGAGTC | 55 | 45 | 20 |
| *OLE2*-gRNA | CGTAAACTATTTTTAATTTGAGGATCGTACGCCCACAACGGTTTTAGAGCTAGAAATAGC | 74.2 | 38.3 | 60 |
| *POX1-3*-gRNA | CGTAAACTATTTTTAATTTGAGAGGTGGACTTCAAAGCAGGTTTTAGAGCTAGAAATAGC | 72.8 | 48 | 60 |

**CRISPR cassette construction**

The CRISPR cassette was formed from three different fragments originating from three plasmids: Cau-pADH99, Cau-pADH110, and Cau-pADH147. The Cas9 cassette was released by digesting 2 μg of Cau-pADH99 with Pmel (New England Biolabs). Cau-pADH110 was amplified using primers AHO1237 and Cau-snR52-1R to form the 5'-end of the gRNA cassette (fragment A). The 3' end of the gRNA cassette was formed by amplifying Cau-pADH147 with a target gene-specific primer that introduced the CRISPR site into the gRNA and Cau-HIS-2R (fragment B). CRISPR sites for *OLE2* and *POX1-3* were identified using the CRISPR tool in Geneious Prime® 2023.0.1 ([www.geneious.com](http://www.geneious.com/)).

CRISPR sites, with a 20bp sequence followed by an adjacent protospacer (PAM) were identified in the open reading frame (ORF). The primer was designed by flanking the CRISPR site sequence (20bp with the PAM sequence removed) with 5' CGTAAACTATTTTTAATTTG 3' complimentary to the snR52 sequence in pADH110 and 5' GTTTTAGAGCTAGAAATAGC 3' complementary to the structural gRNA sequence. The KAPA Taq PCR kit (KAPA Biosystems Inc., 2014) was used to stitch fragments A and B to form fragment C with primers AHO1237 and Cau-HIS-2R. Fragment C was co-transformed with the Cas9 cassette to form an entire CRISPR cassette for integration at the *HIS1* locus, as illustrated in Figure 1.


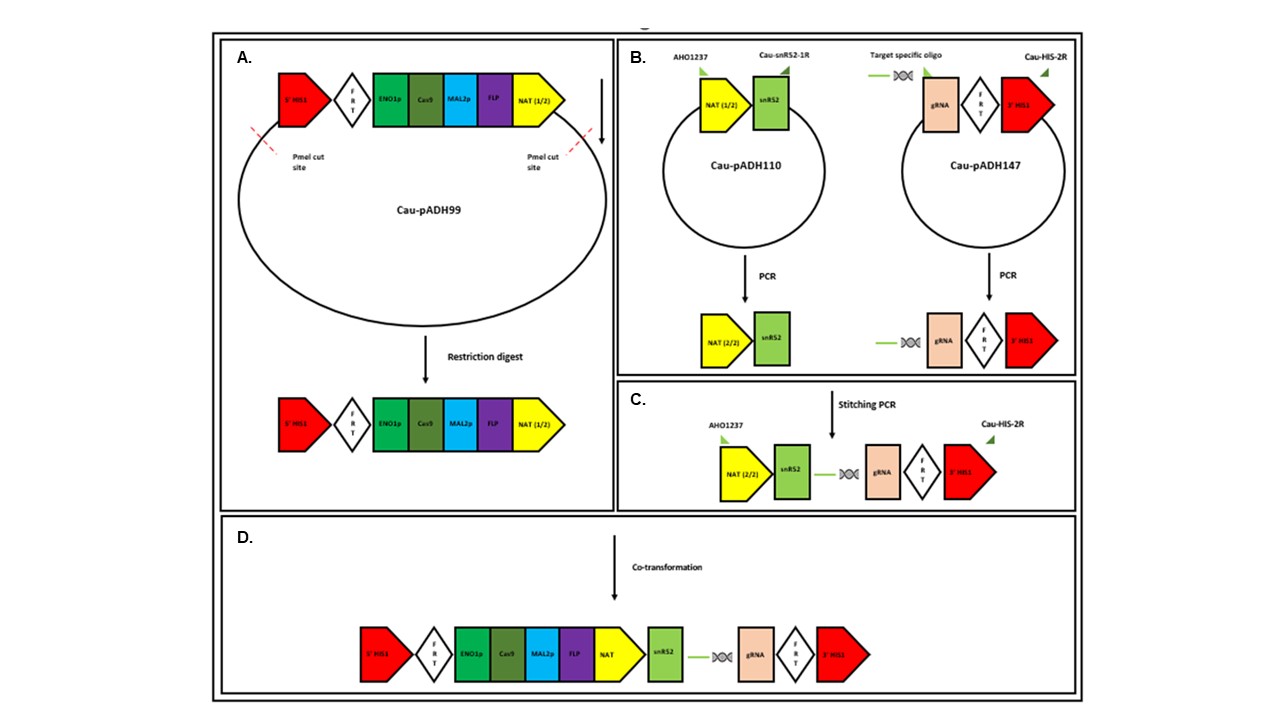


**Figure S1.** Illustration of the CRISPR cassette formation from Cau-pADH99, Cau-pADH110 and Cau-pADH14. **A)** The cas9 cassette was released by digesting pADH99 with Pmel. **B)** The gRNA cassette consisted of two fragments, PCR products of Cau-pADH110 (fragment A) and Cau-pADH147 (fragment B). **C)** The fragments were stitched in a PCR to form the gRNA cassette **D)** The cas9 cassette and the gRNA cassette were co-transformed into *C. auris*. Due to complementary ends in the NAT marker gene, the cas9 and gRNA cassettes were stitched together and incorporated into the *HIS3* genes

**Donor DNA construction for gene editing using CRISPR-Cas9**

Donor DNA (dDNA) was designed to remove the entire open reading frame of the target gene. dDNA consists of two homologous fragments at the 5' (fragment 1) and 3' (fragment 2) ends of the gene of interest. These fragments were PCR-amplified from the genomic *C. auris* DNA. The dDNA consisted of two fragments approximately 1200 bp in length (Table 2). The primers used for fragments 1 and 2 contained overlapping complementary ends. The overlapping sequence was the CRISPR site found in *Arxula adeninivorans*. These two fragments were stitched together using PCR. The dDNA for add-back transformations was the ORF which was amplified from genomic *C. auris* DNA (Figure 2). All PCR reactions were performed using the KAPA Taq PCR kit (KAPA Biosystems Inc. 2014).


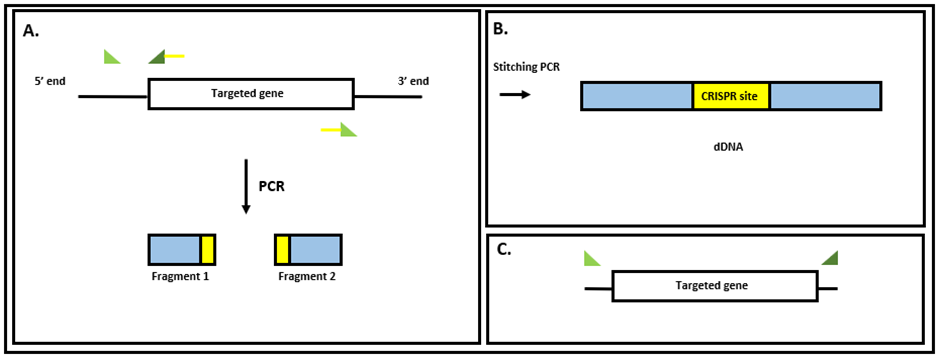


**Figure S2** illustrates the construction of the dDNA for gene manipulation using the CRISPR-Cas9 system. **A)** Two fragments are PCR products of the target genes from genomic DNA, and primers introduce complementary ends. The complementary ends is a unique CRISPR site obtained from *Arxula* *adeninivorans*. **B)** The two fragments were stitched together by PCR. **C)** The dDNA for gene add-back is one fragment, the whole open reading frame (ORF) of the target gene, and PCR of genomic DNA

**Table S2.** List of fragment sizes and primers (1200 bp) used to produce dDNA for deletion of PGE_2_ implicated genes using the CRISPR-Cas9 system.

| Fragment 1 (1200 bp) | | | |
| --- | --- | --- | --- |
| Gene | Forward primer | Reverse primer | Length |
| *OLE2* | *OLE2* -1F (AUX) | *OLE2*-4R CRISPR Site | 1206bp |
| *POX1-3* | *POX1-3* 1F (AUX) | *POX1-3*-4R CRISPR Site | 1293bp |
| Fragment 2 (1200 bp) | | | |
| Gene | **Forward primer** | **Reverse primer** | **Length** |
| *OLE2* | *OLE2*-4F CRISPR site | *OLE2*-1R (AUX) | 1329bp |
| *POX1-3* | *POX1-3*-4F CRISPR Site | *POX1-3* 1R (AUX) | 1360bp |

**Table S3**. List of sizes and primers used to stitch fragments (1200 bp) and produce dDNA for deletion of PGE_2_ implicated genes using the CRISPR-Cas9 system.

| dDNA (Gene deletion with 1200 bp fragments) | | | |
| --- | --- | --- | --- |
| Gene | Forward primer | Reverse primer | Length |
| *OLE2* | *OLE2* -1F (AUX) | *OLE2*-1R (AUX) | 2512bp |
| *POX1-3* | *POX1-3* 1F (AUX) | *POX1-3* 1R (AUX) | 2653bp |

**Table S4**. List of sizes and primers used to produce dDNA for add-back of *ADE2* and PGE_2_ implicated genes using the CRISPR-Cas9 system.

| dDNA (Gene add-back) | | | |
| --- | --- | --- | --- |
| Gene | **Forward primer** | **Reverse primer** | **Length** |
| *OLE2* | *OLE2* -1F (AUX) | *OLE2*-1R (AUX) | 3294bp |
| *POX1-3* | *POX1-3* 1F (AUX) | *POX1-3* 1R (AUX) | 3976bp |

**Removal of the CRISPR cassette**

First, the CRISPR cassette was regenerated to perform add-back mutations in the successful deletion mutants (Figure 3). The deletion mutants were inoculated into 5 mL of YPM (10 g/L yeast extract, 20 g/L peptone, and 20 g/L maltose) and cultivated for 48 h while shaking at 30°C. After incubation, serial dilutions were made, and 10^-6^ dilutions were plated on YPM agar plates and incubated overnight at 30°C. YPM plates were replica plated onto YPD agar supplemented with 600 μg/mL nourseothricin (NAT) (NCT, Jena Bioscience, Germany) and incubated overnight at 30°C. The YPM and YPD-NAT plates were then compared, and colonies that did not grow on YPD-NAT indicated successful removal of the CRISPR cassette. Colonies with removed CRISPR cassettes were selected and used for add-back transformation.


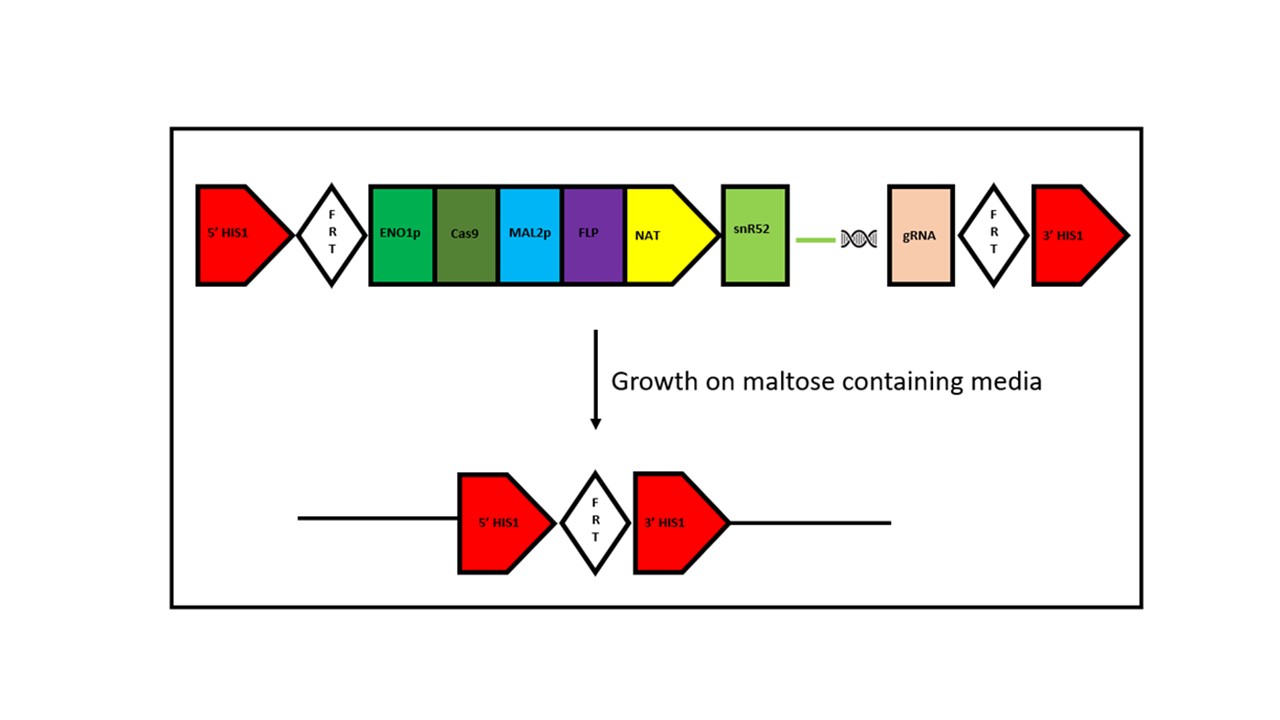


**Figure S3**. Regeneration of CRISPR cassette. Mutant strains were cultivated on maltose-containing media, inducing flippase under the control of a maltose-inducible promoter. The flippase then targets the FRT regions next to the homologous HIS regions, inducing DSB and removing the CRISPR cassette

**Transformation**

*C. auris* was inoculated overnight with shaking at 30˚C in 5mL of YPD media. Next, 100 μL of the overnight culture was inoculated into 5 mL of YPD (ratio 1:50) and incubated at 30°C with shaking until the optical density reached 0.5 – 0.8 at OD_600_. The cells were centrifuged at 1878 × g for 5 min, and the supernatant was removed. The cells were then washed twice with sterile H_2_O by resuspending the cells in 1 mL of sterile H_2_0 and centrifuging for 1 min at 1878 g. After washing, the cells were resuspended in 50 μL water (1/100 of the original volume). The following were mixed with 10 μL carrier DNA (10 mg/mL salmon sperm DNA), 2ug Pmel-digested Cau-pADH99, 50 μL Fragment C, 50 μL DNA, 50 µL washed cells, and 1 mL plate mix (875 μL 50% [w/v]PEG 3350, 100 μL 10 x TE [100 mM Tris, pH 7.5, 10 mM EDTA pH8],25 μL 1 M LiOAc pH7). The suspension was mixed via inversion and incubated overnight at 30°C without shaking.

The cells were then heat shocked at 44.6˚C for 15 min. After the heat shock, the cells were centrifuged for 2 min at 1878 × g, and the supernatant was aspirated. The cells were washed twice in 1 mL of YPD broth and resuspended in 1 mL of YPD. The cells were incubated at 30°C for 4 h. After 4 h, the cells were centrifuged at 2935 × g for 2 min, the supernatant was decanted, and the cells resuspended in residual YPD were left in the tube. The cells were then plated on YPD agar supplemented with 600 μg/mL NAT. YPD-NAT plates were then incubated for 2-3 days at 30°C to allow colonies to form. The resulting colonies were then picked, streaked on YPD-NAT plates, and cultivated overnight at 30°C.

**PCR confirmation of mutants**

Mutants generated through the auxotrophic or CRISPR-Cas9 systems were screened similarly but with different primers (table 22) and PCR conditions, as shown in Tables 17, 18, and 19. Single colonies from the transformation plates were picked, streaked on YPD NAT (600 µg/mL) agar plates, and incubated overnight at 30°C. After incubation, colonies were picked and resuspended in a 1.5 mL microcentrifuge tube containing 100 μL of 200mM LiOAc, 1% SDS solution. The tubes were then incubated for 10 min at 96°C. Then, 300 μL of absolute ethanol was added, and the solution was vortexed. Microcentrifuge tubes containing DNA and cell debris were centrifuged at 15000 g for 3 min to obtain a pellet. The supernatant was then removed, and 70% ethanol was added. The sample was centrifuged at 15000 g for 3 min, and the supernatant was removed. The samples were then dried using a speedy vac. The dried samples were resuspended in 50 μL of ultrapure water and spun down for 30 s to form pellets. For PCR, 2 μL of the supernatant was used for a 25 μL reaction. The KAPA Taq PCR kit was used.

**Results and discussion**

These fragments were PCR amplified from the genomic *C. auris* DNA. The PCR products were run in a gel (Figures 29 and 30)


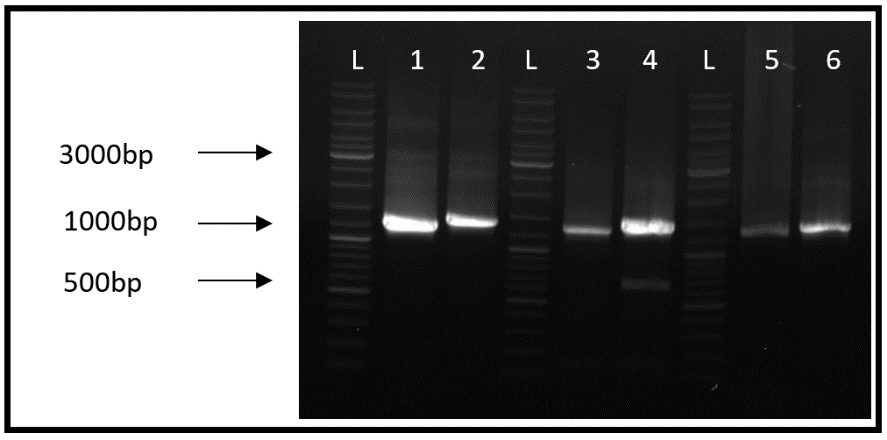


**Figure S4**. Fragments 1 and 2 for deleting PGE_2_-implicated genes. Lanes 1 and 2 represent fragments 1 (1206 bp) and 2 (1329 bp) for *OLE2*. Lanes 3 and 4 represent fragments 1 (1293 bp) and 2 (1360 bp), respectively, for *POX1-3*. The row marked L indicates Fermentas O'GeneRuler^TM^ DNA Ladder


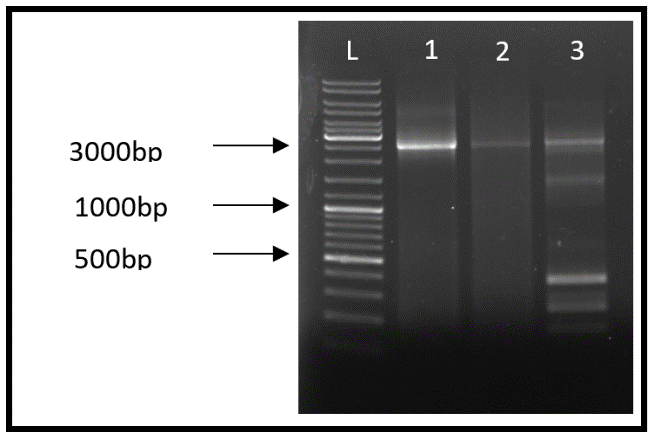
The fragments were then stitched together to form the dDNA in a PCR reaction (Figures 31 and 32).

**Figure S5**. Stitching products of the fragments to form dDNA. Lane 1 represents the dDNA (2512 bp) for *the OLE2* deletion and lane 2 represents the dDNA (2653 bp) for *the POX1-3* deletion. The row marked with L indicates the Fermentas O'GeneRuler^TM^ DNA Ladder.


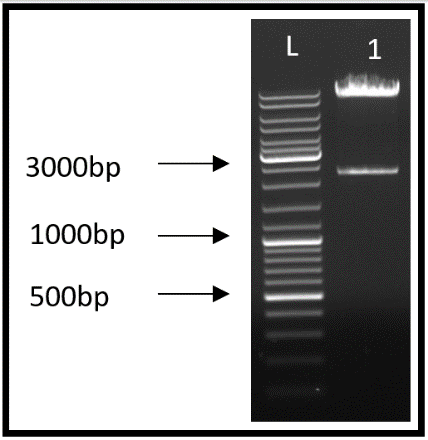
The Cas9 was released by digestion of Cau-pADH99 with Pmel (Figure S6).

L

**Figure S6**. The cas9 cassette was released by digesting pADH99 with Pmel. The digestion products (approximately 8846bp and 2225bp) were run in lane 1. The row marked with L indicates the Fermentas O'GeneRuler^TM^ DNA Ladder.


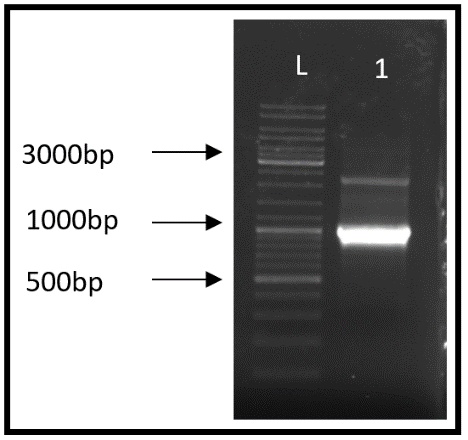
Fragment A (Figure S7) was amplified from Cau-pADH110 with AHO1237, the forward primer, and Cau-snR52-1R, the reverse primer, yielding a fragment of 1317 bp.

**Figure S7**. Fragment A PCR product. Lane 1 represents fragment A (1317 bp) and PCR from Cau-pADH110. The row marked with L indicates the Fermentas O'GeneRuler^TM^ DNA Ladder.


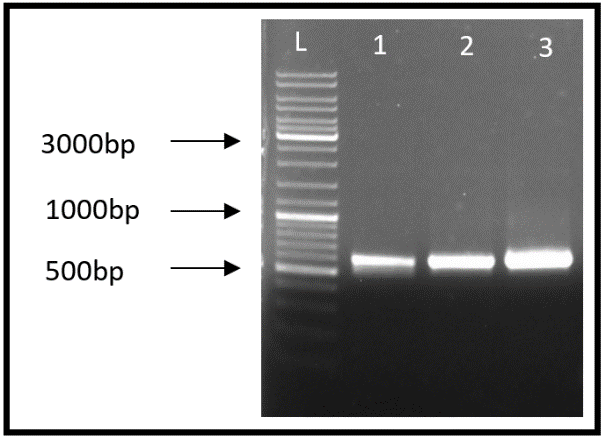
Fragment B (Figure 35) was amplified from Cau-pADH147 using a target gene-specific primer as the forward primer, introducing the CRISPR site into the gRNA cassette, and Cau-HIS1-2R as the reverse primer, yielding a fragment 630bp long.

**Figure S8**. Fragment B (630 bp) for forming the gRNA cassette (fragment C) for PGE_2_ implicated gene deletion. Lane 1 represents fragment B for *OLE2* and lane 3 represents fragment B for *POX1-3* gene deletion. The row marked with L indicates the Fermentas O'GeneRuler^TM^ DNA Ladder.


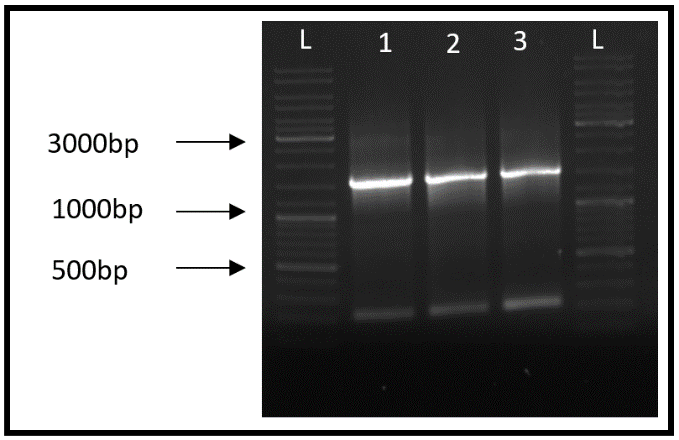
Fragments A and B were stitched (Figure S9) in a PCR with primers AHO1237 and Cau-HIS1-2R.

**Figure S9**. Stitching products of fragments A and B formed fragment C (1947 bp). Lane 1 represents fragment C for *OLE2* and lane 3 represents fragment C for *POX1-3*. The row marked with L indicates the Fermentas O'GeneRuler^TM^ DNA Ladder.


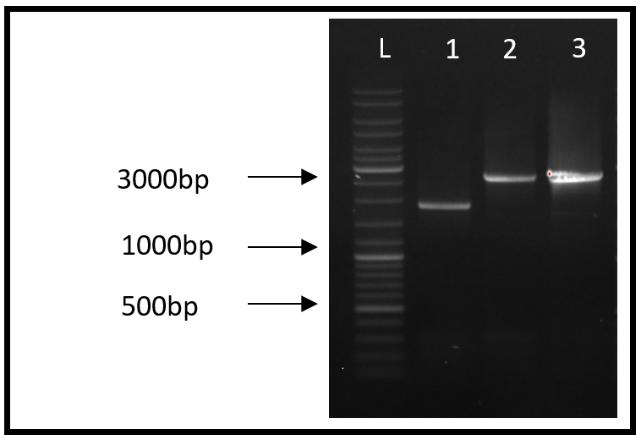
The transformation was performed, and colonies were picked and screened using colony PCR. The *OLE2* mutants for deletion and add-back were screened with *OLE2*-1F(AUX) and *OLE2*-screen (AUX), yielding an 1844 bp fragment for deletion and a 2626 bp fragment for the add-back, and the products were run in a gel (Figure S10). Successful *ole2Δ C. auris* (MRU 293) and *ole2Δ*::*ole2* *C. auris* (MRU 293) strains were generated.

**Figure S10.** The *OLE2* screening results. Lane 1 represents a successful deletion of *OLE2* and the generation of an *ole2Δ* mutant with a length of 1844 bp. Lane 2 represents the add-back of OLE2 and the generation of the *ole2Δ*::*ole2* mutant, which measured 2626 bp. Lane 3 represents the control (2626 bp). Fermentas O'GeneRuler^TM^ DNA Ladder in the L-marked row helped in estimating the sizes of the DNA fragments.


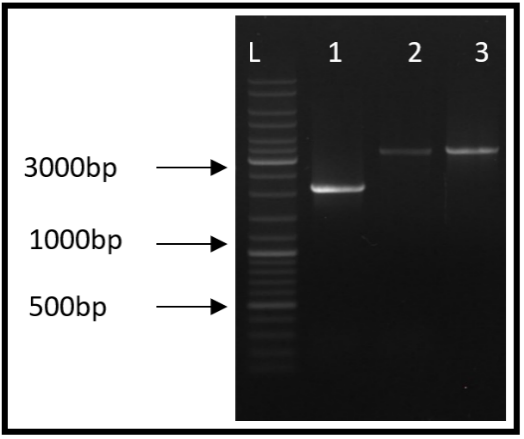
*POX1-3* mutants were screened for *POX1-3* 1F(AUX) and *POX1-3*(AUX), yielding a 2089 bp fragment for deletion and a 3412 bp fragment for deletion add-back. Colony PCR was run on a gel (Figure S11) and showed that successful *pox1-3Δ* C. auris (MRU 293) and pox1-3Δ:: *pox1*-3 *C. auris* (MRU 293) mutants were generated.

**Figure S11**. The *POX1-3* screening results. Lane one represents a positive screening result (2089 bp) for deletion of the *POX1-3* gene, generating a *pox1-3Δ* mutant. Lane 2 represents the add-back of *POX1-3* and the generation of the *pox1-3Δ::pox1-3* mutant, which measures 3412 bp. Lane 3 represents the control (3412 bp). Fermentas O'GeneRuler^TM^ DNA Ladder in the L-marked row helped in estimating the sizes of the DNA fragments.


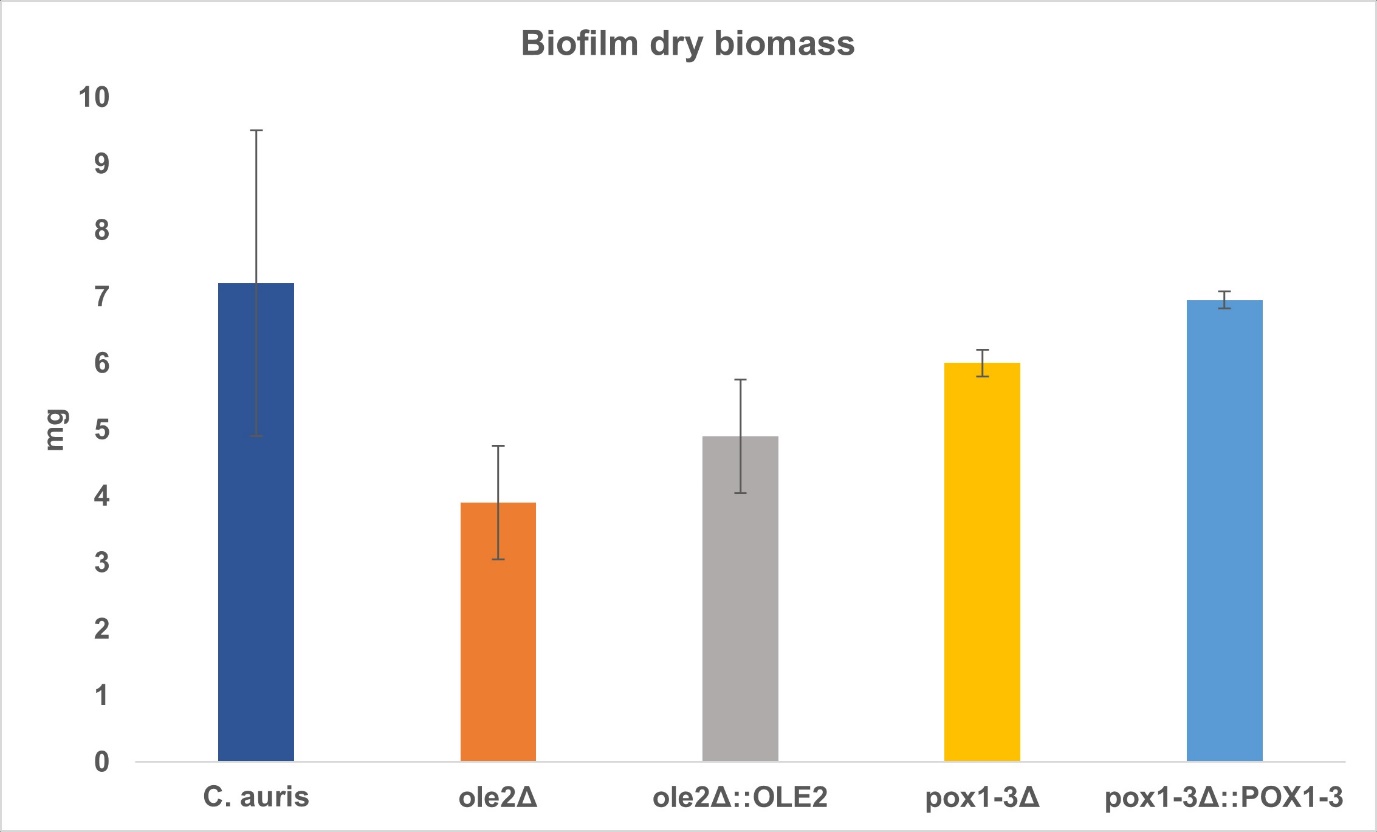


## **Figure S12**. Biofilm biomass produced by the different C. auris strains as described in the Material and Methods section: **Biofilm formation and biomass determination.** This was used to normalise the PGE_2_ concentration.
